# Supplementary material for: In Medio Stat Virtus: Moderate Cognitive Flexibility as a Key to Affective Flexibility Responses in Long-Term HRV
Source: Sensors (Basel). 2024 Dec 17;24(24):8047. doi: 10.3390/s24248047 (PMC11679733; doi:10.3390/s24248047)

Table S1. Descriptives statistics of high and low Cognitive Flexibility Groups.

	Group	N	Mean	SD	SE
PRE_SDNN (ms)	HIGH	12	52,7677	16,91221	4,88213
	LOW	11	47,9745	20,91124	6,30498
PRE_SD HR (bpm)	HIGH	12	4,5953	1,29662	0,3743
	LOW	11	4,2068	1,95562	0,58964
PRE_LF_HF_ratio_AR	HIGH	12	2,7782	2,41336	0,69668
	LOW	11	3,2928	6,30438	1,90084
PRE_SD2_SD1_ratio	HIGH	12	2,2285	0,64825	0,18713
	LOW	11	2,0169	0,79812	0,24064

Table S2. Bayesian Independent Samples T-Test

	BF_{01}	error %
PRE_SDNN (ms)	2,308	0,00182
PRE_SD HR (bpm)	2,348	0,00179
PRE_LF_HF_ratio_AR	2,572	0,00162
PRE_SD2_SD1_ratio	2,209	0,00191

Table S3. Inferential plots with Bayes Factor Robustness Check

PRE_SDNN (ms)

Prior and Posterior

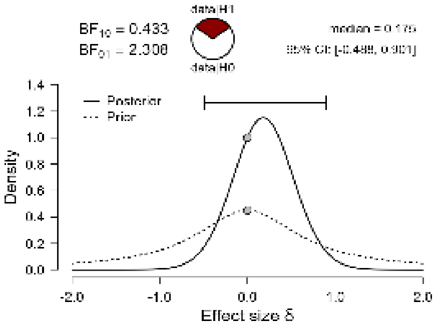

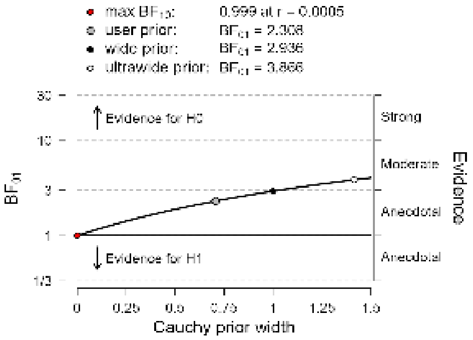

PRE_SD HR (bpm)

Prior and Posterior

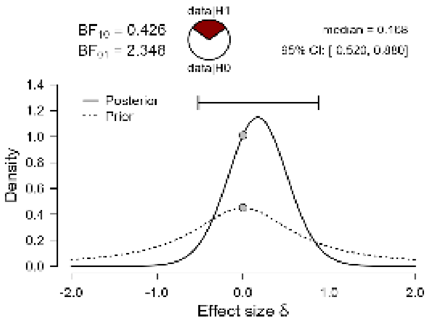

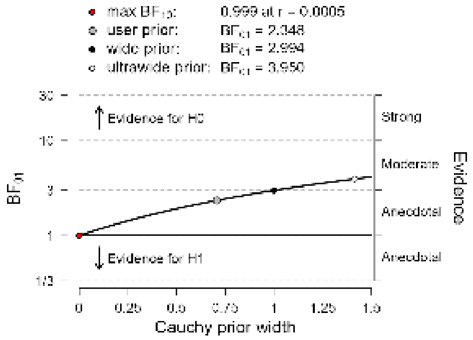

PRE_LF_HF_ratio_AR

Prior and Posterior

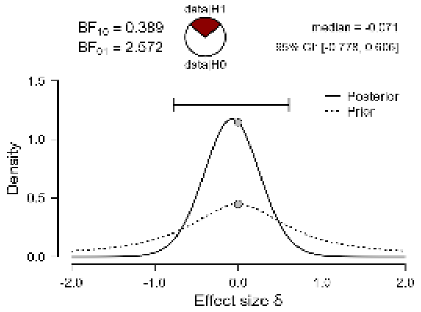

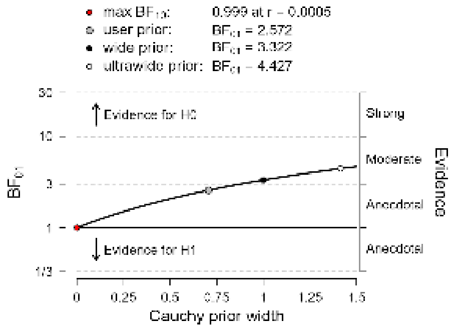

PRE_SD2_SD1_ratio

Prior and Posterior

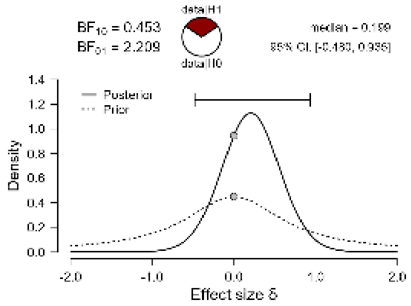

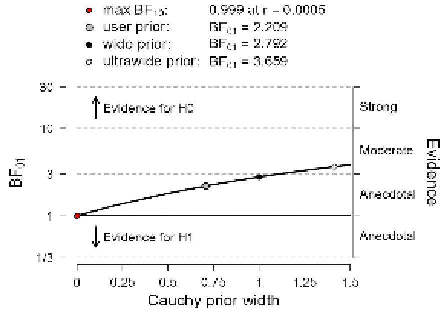

Supplement: Supplementary file 1 [file sensors-24-08047-s001.zip › sensors-3321750-supplementary.pdf]
